# Pre-symptomatic intervention for autism spectrum disorder (ASD): defining a research agenda

**DOI:** 10.1186/s11689-021-09393-y

**Published:** 2021-10-15

**Authors:** Rebecca Grzadzinski, Dima Amso, Rebecca Landa, Linda Watson, Michael Guralnick, Lonnie Zwaigenbaum, Gedeon Deák, Annette Estes, Jessica Brian, Kevin Bath, Jed Elison, Leonard Abbeduto, Jason Wolff, Joseph Piven

**Affiliations:** 1 Carolina Institute for Developmental Disabilities, University of North Carolina, Chapel Hill, NC USA; 2 Program for Early Autism Research Leadership and Service (PEARLS), University of North Carolina, Chapel Hill, NC USA; 3 Department of Psychology, Columbia University, New York, NY USA; 4 Center for Autism and Related Disorders, Kennedy Krieger Institute, Baltimore, MD USA; 5 Department of Psychiatry and Behavioral Sciences, The Johns Hopkins University School of Medicine, Baltimore, MD USA; 6 Division of Speech and Hearing Sciences, University of North Carolina, Chapel Hill, NC USA; 7 Center on Human Development and Disability, University of Washington, Seattle, WA USA; 8 Department of Pediatrics, University of Alberta, Edmonton, Canada; 9 Department of Cognitive Science, University of California, San Diego, San Diego, CA USA; 10 Department of Speech and Hearing Sciences, University of Washington Autism Center, University of Washington, Seattle, WA USA; 11 Holland Bloorview Kids Rehabilitation Hospital, Toronto, Canada; 12 Department of Paediatrics, University of Toronto, Toronto, Canada; 13 Department of Neuroscience, Brown University, Providence, RI USA; 14 Institute of Child Development, University of Minnesota, Minneapolis, MN USA; 15 University of California, Davis, MIND Institute, University of California, Davis, Sacramento, CA USA; 16 Department of Educational Psychology, University of Minnesota, Minneapolis, MN USA

## Abstract

Autism spectrum disorder (ASD) impacts an individual’s ability to socialize, communicate, and interact with, and adapt to, the environment. Over the last two decades, research has focused on early identification of ASD with significant progress being made in understanding the early behavioral and biological markers that precede a diagnosis, providing a catalyst for pre-symptomatic identification and intervention. Evidence from preclinical trials suggest that intervention prior to the onset of ASD symptoms may yield more improved developmental outcomes, and clinical studies suggest that the earlier intervention is administered, the better the outcomes. This article brings together a multidisciplinary group of experts to develop a conceptual framework for behavioral intervention, during the pre-symptomatic period prior to the consolidation of symptoms into diagnosis, in infants at very-high-likelihood for developing ASD (VHL-ASD). The overarching goals of this paper are to promote the development of new intervention approaches, empirical research, and policy efforts aimed at VHL-ASD infants during the pre-symptomatic period (i.e., prior to the consolidation of the defining features of ASD).

## Background

The prevalence of ASD has increased over the last decade and is currently estimated to be present in 1 in 54 school-age children [1]. Early detection and intervention for ASD provide an opportunity to foster development, ultimately improving the quality of life and decreasing the lifetime financial and mental health costs associated with ASD. Access to evidence-based interventions early in life may also mitigate the elevated levels of stress, anxiety, and depression experienced by caregivers of individuals with ASD [2, 3]. The total annual costs of caring for children with ASD may exceed 461 billion dollars by 2025 in the USA alone [4]. The lifelong financial costs associated with maximizing functional and quality of life outcomes for those with ASD highlights the need for research efforts into very early intervention [5].

Progress has been made toward developing and providing interventions during the early years; however, the efficacy of these interventions in altering developmental trajectories is typically small to moderate [6, 7]. Identifying infants at very high likelihood of developing ASD (VHL-ASD) prior to symptom emergence provides a novel opportunity to implement intervention during a potentially more sensitive window, ultimately maximizing outcomes. Concurrently, research is growing to support the identification of behavioral and neurobiological markers that indicate elevated likelihood of ASD as early as 6 months of age, well before the onset and consolidation of ASD symptoms into a diagnosable condition [8–12]. This provides a novel opportunity, and ethical imperative [13], to examine the efficacy of intervention before full symptom onset (referred to throughout as a pre-symptomatic intervention; see Table 1) when the brain is maximally malleable, potentially yielding more robust improvements in functional outcomes than could be achieved with intervention that begins after consolidation of symptoms into diagnosis [15]. This article therefore presents a proposal for innovative research focused on the period before consolidation of symptoms into a diagnosis, in the latter part of the first and second years of life, and is aimed at initiating a comprehensive conversation in the field on pre-symptomatic intervention for ASD that will stimulate future research on this potentially important period of development and opportunity for intervention.

**Table 1:** Defining “pre-symptomatic” and “very high likelihood”

**Pre-symptomatic** • This work emphasizes the development of intervention prior to 12 months of age, before the consolidation of symptoms into a diagnosable disorder. • The term “pre-symptomatic” is used throughout the manuscript to refer to the postnatal period until ~ 12 months of age—a period when no or very few symptoms of ASD are present, and symptoms have not consolidated into a diagnosable disorder (ASD). • Alternative terminology, including “pre-diagnosis”, “pre-emptive”, “pre-syndromic”, and “prodromal” was considered, though these options were eliminated as none was considered a better option with respect to criteria of accuracy, specificity, and conventionality. **Very high likelihood (VHL)** • High likelihood (HL) is defined by the increased likelihood of a later ASD diagnosis based on one “risk” marker, such as having a sibling with ASD. • Very high likelihood of ASD (VHL-ASD) refers to a combination of “risk” markers: such as predictive brain markers and a sibling with a diagnosis of ASD. Defining VHL-ASD may be expanded to include other combinations of markers as research advances in its accuracy and reproducibility of predicting later ASD. • The term “likelihood” is used throughout, though “risk” has been used in prior empirical work. Further discussion within the field surrounding the use of this term is necessary [14]. • Developing a framework for pre-symptomatic intervention in VHL-ASD infants is the primary focus of this work as it holds great importance to the field and relevance to a significant number of families with children born after the birth of an autistic child. • In the future, this work can be extended to the general population of autistic children, though to date, biomarkers have not been identified to make this currently possible.	

### Goals of this work

This paper will provide the conceptual framework necessary for identifying infants who might benefit from pre-symptomatic intervention, as well as initial intervention targets and methodologies that research can build upon. We focus this piece on very high-likelihood ASD (VHL-ASD) infants, defined as having dual risk factors of neuroimaging markers of ASD and a positive family history of ASD. The term “likelihood” is used throughout, rather than “risk”, though further discussion within the field surrounding language is necessary [14]. We expect that this stringent designation for likelihood in initial pre-symptomatic intervention research will reduce the probability of exposing infants to unnecessary clinical care and maximize the probability that those who need care most are likely to receive it.

The pre-symptomatic period is generally considered the time before and during the emergence of core symptoms of ASD in the latter part of the first and second years of life, that typically consolidate into a clinical diagnosis of the behavioral syndrome of ASD, around 24 to 36 months of age [1]. Here, we (1) outline the growing literature on biological and behavioral precursors to ASD that will facilitate the identification of VHL-ASD infants and (2) provide a conceptual foundation for the development of pre-symptomatic interventions for ASD in infants starting at 6 months of age, with a focus on precursors to an ASD diagnosis. The conceptual framework for a pre-symptomatic intervention draws from empirical literature supporting the identification of VHL-ASD infants by virtue of sibling status combined with neuroimaging markers, though this has not yet been implemented to identify a population for treatment. When considering the implications of identifying VHL-ASD infants, MacDuffie et al. [13] propose two strategies: (1) initiate extra monitoring and begin intervention at first onset of symptoms or (2) initiate a pre-symptomatic intervention. This paper focuses on the latter approach.

As the ability to accurately identify VHL-ASD infants improves, researchers and clinicians are left with a number of critical questions:

1. How can improved outcomes in VHL-ASD infants be promoted?
2. Is additional monitoring and screening without explicit intervention what families want?
3. Should we wait until symptom onset before initiating intervention?
4. If we initiate intervention prior to the onset of symptoms, what do we target?
5. Given that the majority of research on behavioral interventions in ASD has been conducted following the consolidation of symptoms into a diagnosable condition, how do we conceptualize pre-symptomatic intervention to limit the impact of impairing symptoms that are not currently present or are present in only their earliest manifestations?
6. How do we conceptualize interventions aimed at presumed precursors of later ASD that might disrupt downstream cascading brain and behavior changes leading to ASD-associated impairments, as some have suggested [16, 17]?
7. What are the ethical and social implications of pre-symptomatic identification and intervention?

These questions are becoming increasingly relevant as early biomarkers are identified and refined. Although we cannot fully address all of these questions in this article, we hope this discussion will promote research and discourse into pre-symptomatic interventions for ASD. The ideas proposed in this article are based on the collective expertise of a multidisciplinary group that convened in 2020, and in an ongoing fashion throughout the writing of this paper. This group included experts in multiple disciplines including developmental disabilities, typical infant development, infant and child cognition, behavioral and brain development, learning processes, early interventions (for ASD, neurodevelopmental disorders, and nonclinical populations), psychology, psychiatry, pediatrics, neurobiology, preclinical trials, and ethics.

### Detection of early ASD symptoms and early intervention post-diagnosis

Autism-specific early intervention is typically initiated after a child has received a diagnosis of ASD. The diagnosis is rendered once a child meets a threshold of defining behavioral symptoms and associated impairment, as delineated by the DSM-5 [18] or ICD-11 [19]. The average age that a child receives an ASD diagnosis is 4 years in the USA, with nearly half of children diagnosed after entering elementary school and after early intervention services could be implemented [20]. Lowering the age of diagnosis to two or three, while important, may not substantially improve outcomes. Nahmias et al. [6] reported that early intervention is administered to toddlers with ASD, current approaches appear to yield only modest impacts, at best, on functional outcomes, particularly when implemented in the community (i.e., effect sizes of *d* = 0.2–0.3). The modest effects of interventions with toddlers with ASD highlight the importance of studying interventions implemented during an earlier, potentially more sensitive window. Advances in earlier detection and diagnosis provide opportunities to develop and evaluate the efficacy of pre-symptomatic interventions.

### Identifying very-high-likelihood (VHL-ASD) infants using behavioral and biological markers

Infant siblings, who have a genetically increased likelihood of developing ASD (HL) by virtue of having an older sibling with ASD, have been the focus of numerous prospective studies tracking the emergence of ASD symptoms in infancy [21–24]. Approximately 20% of siblings go on to receive a later diagnosis of ASD, highlighting the limited cost-effectiveness of current practices of intervening with all HL siblings [21]. Identifying behavioral or biological markers that can accurately predict those that go on to receive a diagnosis of ASD is therefore essential to feasibly and cost-effectively identify candidates for intervention prior to symptom onset and/or consolidation of symptoms into a diagnosis. Unfortunately, HL infants who go on to develop ASD are often behaviorally indistinguishable from those who do not develop ASD [10, 25, 26]. A number of studies have demonstrated differences between HL and low-likelihood (LL) infants [27–34]. While they have revealed group differences between those who go on to develop ASD and those HL infants who do not, these behavioral measures have not, as yet, provided clinically actionable positive predictive values (*PPV*) on an individual level, that would suggest cost-effectiveness of what are typically costly interventions. Only a few of these studies have identified behavioral differences between HL-positive and HL-negative groups [34, 35]. For example, Estes et al. [34] found that HL-positive cases had lower cognitive abilities in infancy compared with the HL-negative, and Elison et al. [35] found lower social fixation in an eye tracking task at 6 months of age for HL positive versus HL negative. While neither of these studies examined individual-level prediction, a separate project used a data driven approach that included both a training and a validation sample at 18 months of age, to predict on an individual level based on ASD symptoms measured by the Autism Diagnostic Observation Schedule and the Autism Diagnostic Interview, Revised (ADI-R; [36]). This work yielded a positive predictive value (*PPV*) for a 36-month ASD diagnosis (18 months later) of only 50% [37], which is insufficient for guiding cost-effective decisions regarding intervention on an individual level. As such, additional predictive characteristics may assist in identifying individuals at very high likelihood (VHL-ASD) of developing ASD. While individual diagnostic prediction based on behavior is limited, it is possible that early behavioral differences inform a conceptual framework for a prodromal period before the consolidation of ASD symptoms. This period (specifically between birth and 12 months) may be ripe for pre-symptomatic interventions that target these early behaviors to avert subsequent cascading events leading to the impairing outcomes associated with ASD [17].

Biological markers for identifying very early individual-level prediction are promising. Several recent MRI studies suggest that brain imaging provides clinically useful, pre-symptomatic classifiers in infants as young as 6 months that accurately predict later diagnosis. Employing a data-driven, deep learning strategy, Hazlett et al. [8] reported that a classifier derived from a combination of structural MRI scans from infants at both 6 and 12 months of age was able to accurately predict, at an individual level, later diagnosis of ASD in eight out of ten subjects (*PPV* = 0.80); though the small sample size precludes confidence about reliability or generalizability, these results are encouraging. Consistent with these findings, Emerson et al. [38] found that a data-driven approach using data from functional connectivity resting state MRI (fcMRI) at 6 months of age was highly accurate in predicting later individual ASD diagnoses at 24 months of age.

Using machine learning from prenatal anatomical ultrasounds, Caly et al. [39] found an individual-level *PPV* of 0.77 for infants with a later diagnosis of ASD versus non-ASD familial siblings or typically developing infants. A recent study used machine learning to identify a subgroup of children with ASD (*n* = 450; vs. typically developing children; *n* = 342) using maternal autoantibody patterns; researchers reported 100% accuracy with this method [40], though the specificity of this method is yet to be confirmed with HL non-ASD samples. Taken together, these reports replicate the idea that there exist pre-symptomatic biomarkers for prediction of later ASD, in particular through brain imaging. We note however that a large-scale HL infant sibling study, using multimodal MRI to attempt to replicate findings from Hazlett et al. [8] and Emerson et al. [38], is currently underway.

While the concept of pre-symptomatic (VHL-ASD) detection of neurodevelopmental disorders is relatively new, the idea is not new in other areas of neurobiological medicine. For example, it is well-known that prior to a diagnosis of Parkinson’s disease, affected individuals have substantial loss of dopamine neurons in the substantia nigra [41]. Similarly, changes in brain function on PET imaging have been detected years before the onset of cognitive impairment [42]. Once symptoms emerge and consolidate into a diagnosis of Parkinson’s and Alzheimer’s diseases, considerably more substantial brain changes are present, further complicating as well as diminishing optimism regarding intervention [43, 44].

**Fig. 1:**
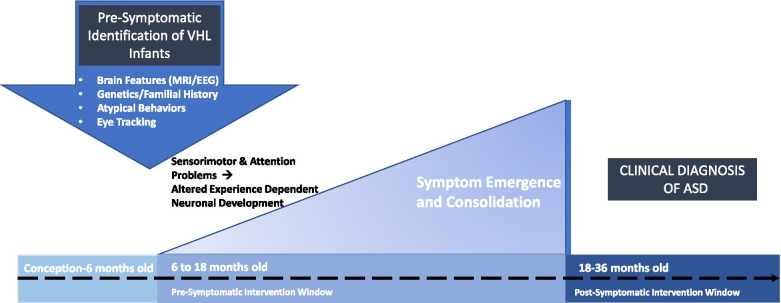
Conceptual framework for pre-symptomatic intervention

Results from the predictive studies of Hazlett et al. [8], Emerson et al. [38], and Caly et al. [39] suggest that leveraging biomarkers of later ASD may be a feasible method for early identification of VHL-ASD infants*.* Frazier et al. [45] proposed combining ASD screening measures (not yet clinically employed), such as saliva testing (polyomic RNA markers [46]) with eye tracking, to identify children who will later receive an ASD diagnosis. Similar to this screening approach, combining multiple HL identification methods may provide a feasible way to identify VHL-ASD infants who can receive a very early pre-symptomatic intervention. Currently, VHL-ASD infants could be identified using the combination of ASD-positive sibling status and the presence of neuroimaging markers [8]. In the future, more individual-level biomarkers may be identified, allowing for further combination of relevant indicators. However, identifying a group of siblings that possess a predictive brain marker is immediately possible and offers an opportunity to deliver treatment to a significant subpopulation. While it is possible that this method of identification could be applicable to broader HL populations, we suggest a more narrowed subject pool to maximize the feasibility of initial efforts toward developing a pre-symptomatic intervention. Yet, in a review of the financial burden of acquiring early brain biomarkers, Williamson et al. [47] make a strong case that such a test would, in fact, be highly cost-effective, especially if an intervention could be implemented. Additional cost-effectiveness may be found when considering the utility of prediction based on prenatal screening that could be integrated into standard care [39]. Prenatal screening could provide the opportunity to prepare tailored interventions as soon as possible. The ethical implications of infant prediction highlight the need to provide families with intervention options at the point these identification techniques are implemented [48, 49]. As pre-symptomatic prediction methods increase in accuracy, there is a critical ethical need to establish a research agenda for the development of pre-symptomatic intervention (Fig. 1).

### Providing a framework: “development as adaptation”

The design of a pre-symptomatic intervention presupposes a non-deterministic or plastic developmental trajectory that has the potential to be altered (e.g., via environmental modifications), leading to cascading effects that yield different, more adaptive outcomes. Within ASD, the growing capacity to identify VHL-ASD infants provides a potential opportunity to favorably alter an infant’s developmental trajectory during a more sensitive window, leading to improved functional outcomes, decreased service needs, and higher quality of life. It is possible that pre-symptomatic interventions may lead to immediate benefits, such as increased social awareness, responsivity, and decreased maladaptive behaviors. It is also possible that, while immediate functional benefit is not apparent, later functional outcomes may be enhanced, highlighting the need for long-term follow-up and evaluation.

The developmental trajectory of a child is considered to be the result of their ability to process and adapt to the environment in light of their unique predispositions (e.g., genetics). This concept of “development as adaptation” has been applied to ASD by Johnson et al. [50]. According to this view, adaptation is the result of learning which information in the environment is useful for surviving and/or thriving. However, this adaptation is not necessarily adequate for thriving in future environments.

Johnson et al. [50] discuss several developmental principles relevant to the design of a pre-symptomatic intervention: redundancy, reorganization, niche construction, and timing. With respect to learning environmental patterns, multiple (“redundant”) neural systems can gather information at once. Provided the same environment, different children may use different attention, sensory, or memory systems to gather information. As a result, each child may extract unique information that influences what is learned and thereby impacts subsequent learning. *Redundancy* permits compensation for impoverished input to one system by employing other systems to promote alternative developmental trajectories by learning alternative elements of multidimensional events. *Niche construction* describes differential development of the neural pathways involved in learning based both on the necessity and the *efficacy* of what is learned. As different neural circuits are employed, developmental changes “reorganize” the function of these circuits *and* the functions of other neural regions to which the circuits are strongly connected through a process known as *reorganization*. Developmental trajectories can also be influenced by the *timing* of learning. Indeed, the vast literature on critical and sensitive windows highlights the times during which the brain is most adaptable to learning (e.g., aspects of vision [51];). However, when early learning is not efficient for later needs, timing can shift to compensate, and learning of necessary information may occur at different times according to a child’s needs and sensitivities. For example, although there is a sensitive period for aspects of vision, those systems remain somewhat amenable to training, even in adulthood [52].

This conceptual framework of “*development as adaptation*” is critical to understanding the ASD phenotype. Rather than viewing ASD as a set of behaviors deviating from normative expectations with predetermined outcomes, this framework considers the behavioral phenotype of ASD to develop as a consequence of the interplay of predispositions (e.g., genetic, epigenetic, and prenatal factors) and environmental learning that is continually adjusted by the child’s emerging capacities and challenges. This framework also provides a lens through which to consider early pre-symptomatic intervention for ASD and highlights the opportunity to modify, or enhance, an infant’s environment to promote the best possible trajectory based on the principles of redundancy, niche construction, reorganization, and timing. The foundation of a pre-symptomatic intervention discussed in this article lies in this conceptual framework and is underscored as environmental modifications are proposed.

### Post-diagnosis early interventions inform pre-symptomatic intervention

Early intervention for ASD predominantly focuses on children between 2 and 5 years of age, who have already received an ASD diagnosis. These interventions have used a variety of approaches, including methods based in operant conditioning (e.g., applied behavior analysis; ABA) with a focus on skill building, usually in highly structured, adult-led contexts, and naturalistic developmental behavioral interventions (NDBIs; [53]) with an emphasis on embedding intervention in meaningful, dyadic engagement contexts [54]. Both of these approaches rely on modifying the child’s environment to promote learning skills to optimally adapt and thrive. Results of these interventions have been mixed. ABA methods have been moderately effective in improving IQ, adaptive outcomes and interfering behaviors [55–59] but outcomes indicate that only 20–50% of children show improvement [60]. Improvements from these interventions might be linked to intervention intensity or implementation method, but this relationship is not clear [61, 62].

Caregiver-mediated NDBIs, which often focus on fostering caregiver responsivity, have shown some impact on caregiver–child engagement and subtle improvement in child receptive language and ASD symptom severity [7, 63–66]. Some research indicates that NDBIs that include caregiver-mediated in addition to clinician-led components, may be the most advantageous [62, 67, 68], though a follow-up and recent meta-analysis did not find an association between effect sizes and implementer (caregiver-mediated, teacher/clinician-led, combination [61]). In 2- to 5-year-olds who received a parent-mediated, communication-focused intervention improved parent–child behavioral synchrony was noted after 13 months [69]. These improvements as well as lower ASD severity were reported up to 5 years [70]. Despite these results, community-based early intervention has had a modest impact at best, with small effect sizes reported [6]. A systematic meta-analysis showed that in randomized controlled trials (RCTs) that accounted for detection bias (e.g., assessors were blind to group membership), no significant effects were found across a range of implementation strategies and outcome types [7]. Pre-symptomatic identification provides a novel opportunity to implement caregiver-mediated intervention during a potentially more sensitive time window in the first year of life.

While the limited efficacy of early interventions may be disappointing, the feasibility and acceptability of these interventions is promising. Early interventions, which most often include a caregiver component, have been shown to be feasible with young children and can be implemented with a range of intensities [66, 69, 71]. Caregivers are able to implement intervention techniques with fidelity, parents report feeling empowered [66, 72] and describe positive results of intervention (even when objective markers of improvement are null) [71, 73]. As such, we have evidence that early intervention can be feasibly implemented with high fidelity with caregivers who are accepting of integrating these techniques into their daily lives and find these interventions to be helpful. There is a possibility that a pre-symptomatic intervention (e.g., before 12 months of age) that is built upon the feasible and acceptable foundation of post-diagnosis intervention, integrating novel developmental intervention targets, will be more effective and yield more optimal functional outcomes than a post-diagnosis caregiver-mediated intervention.

### Preliminary studies of pre-symptomatic intervention for ASD

Only a handful of RCTs have attempted intervention with pre-symptomatic HL infants, some with siblings of children with an ASD diagnosis, and some identified through early screening or referrals. Because these studies are populated primarily by siblings of an ASD individual and only 20–30% of these subjects will receive a later diagnosis of ASD, the sample sizes limit the power and subsequent interpretation. Furthermore, these RCTs have found few significant post-intervention main effects; effect sizes generally have been small, although feasibility, acceptability, and implementation fidelity were high [74–78]. In trials that included follow-up periods, there is limited evidence of diverging trajectories between groups over time [71, 79]. More encouragingly, two trials have yielded evidence that pre-symptomatic caregiver-mediated intervention has an indirect effect on child outcomes that are mediated through changes in caregiver responsiveness [77] or the extent to which caregivers implement the intervention strategies [80]. A recent RCT implemented an intervention between 12 and 18 months of age; while no immediate treatment effects were found (after about 6 months of intervention), long-term improvements (by three years of age) were found in parent responsiveness and language child outcomes [81]. However, only a small number of children (*n* = 12) received a diagnosis of ASD at 3 years of age, highlighting the limited power to interpret these results. These data suggest that the hypothesized mechanisms of change are operating as intended (i.e., producing caregiver-mediated effects). Intervention with VHL-ASD infants identified by a combination of markers, as proposed here, has not yet been investigated. It is possible that in VHL-ASD infants who begin to receive early intervention very young (e.g., 6 months of age), effectiveness may be enhanced.

### Is intervening earlier better? Evidence from preclinical studies

Insights gleaned from animal models indicate that development and timing of intervention are critical considerations when dissecting the pathophysiology of neurodevelopmental disorders [82]. Mouse models of monogenic disorders associated with ASD, such as Rett syndrome and Angelman syndrome, provide a fertile testing ground to understand the impact of genetic or environmental variables on neural and circuit development, behavioral markers of ASD pathology (e.g., endophenotypes), and efficacy of interventions for remediation of endophenotypes. Nevertheless, rodent models are limited due to the importance of language and complex communication phenotypes as defining features of ASD. The examples below are provided, while acknowledging these limitations, to demonstrate proof of principle, as illustrated in relevant animal models.

Across the developed models, a number of attempts have been made to limit the development of behavioral markers in mice. Recent work in the *nlgn3* knockout (KO) model of ASD has shown that early postnatal restoration of *nlgn3* expression as well as novel pharmacological interventions that target gene translational processes associated with oxytocin signaling during the preadolescent period are capable of rescuing social deficits [83]. Similarly, work in the CNTNAP2 model of ASD has shown altered neurodevelopmental trajectories arising from gene deletion [84] and development of ASD-like endophenotypes that could be rescued by modulation of pathways impacting oxytocin signaling. In these studies, intervention was most efficacious when implemented during the early postnatal period [85].

In the mouse line genetically designed to model Rett syndrome, behavioral intervention during the pre-symptomatic period dramatically improved the performance of motor and memory tasks and significantly delayed symptom onset; however, intervention beyond the pre-symptomatic period had no impact. Moreover, this research found an association between behavioral intervention and specific neuronal development and activation [86]. In the model of Angelman syndrome, genetic strategies have been developed to investigate timed reinstatement of gene expression in mice lacking the *UBE3A* gene on neurodevelopmental outcomes. Only juvenile reinstatement of gene expression could rescue anxiety-like behavior, marker tasks for repetitive behaviors, and risk for epilepsy [87]. Similarly, if *UBE3A* gene expression is intact throughout early development and is only deleted following the juvenile period, there are limited effects on behavior [88]. Most recently, *CRISPR-Cas9* gene therapy was used to “un-silence” the dormant paternally inherited *UBE3A* allele during the prenatal and early postnatal period, rescuing the expected anatomical and behavioral phenotypes associated with Angelman syndrome [89].

The importance of developmental timing on risk for symptom development appears to be present for other genetic conditions as well. For example, the phenotype of *CNTNAP2* mice has been partially rescued through optogenetic manipulation of excitation/inhibition balance in the prefrontal cortex [90], pointing to possible effects of this gene on regional brain development and signaling. Work in fragile X syndrome model mice has been effective in developing multiple pharmacological and genetic rescue strategies for ASD-associated symptoms [91], including modulation of the mGluR5, GABA_B_, CB1, and other targets, although translation of these findings in humans have not yet proven to be successful. Overall, these preclinical trials highlight the potential for very early intervention that can be maximally effective when implemented during a sensitive developmental window, before significant symptom consolidation.

### Timing matters: targeting foundational developmental processes

In human longitudinal studies, VHL-ASD infants often display gradually increasing, persistent lags in development; this often represents a failure to keep up with same-age peers over time, although occasionally explicit loss or regression in skills is observed [92, 93]. Failure to keep up with same age peers is a common phenomenon affecting other risk and disability groups such as infants born preterm. In these other populations, there is evidence that very early interventions minimize this developmental phenomenon [94]. The network of follow-up and intervention programs for infants born preterm/with low birth weight provides a valuable model to consider for VHL-ASD infants, particularly because preterm/low birth weight infants are at an elevated likelihood of developing ASD [22]. In these programs, interdisciplinary professionals provide periodic follow-up assessments and advice subsequent to hospital discharge, making recommendations for a range of child-specific or family-based intervention services if needed. This context has also served as a framework for more comprehensive and intensive preventive intervention programs, often focusing on children highly likely to experience developmental delays as a consequence of the co-occurrence of both biological and environmental risk factors [95].

Of particular relevance is the Play and Learning Strategies (PALS) intervention program for preterm/low birth weight infants [96]. This relatively low-intensity home-based intervention is typically implemented during the time period (6–18 months) that VHL-ASD infants may be potentially identifiable and interventions initiated. Research has documented substantial benefits as a consequence of participation in the PALS intervention for both children and families. For infants receiving intervention, development proceeded at a faster pace with increases evident in many domains including social communication and complex play skills. Mediation analyses suggested that elements associated with caregiver-sensitive responsiveness were primary factors influencing the development of child skills during this sensitive developmental period.

Consistent with PALS, many intervention programs implemented before the second year of life emphasize developmental mechanisms that support the establishment of a high level of caregiver–child synchrony [97]. The PALS curriculum has many overlapping features with early interventions for ASD, including iBASIS (parent-mediated video-aided intervention) and Promoting First Relationships (PFR) [79, 98]. There is evidence from clinical trials to suggest that earlier intervention (e.g., prior to 48 months of age) can impact outcome [99], although results are mixed [59, 61]. Pre-symptomatic interventions for VHL-ASD children can build on this work from other high-likelihood infant populations that demonstrate the utility of family resources and caregiver–child synchrony on child outcomes.

### Environmental targets that maximize plasticity

Early brain differences associated with ASD may impede experience-dependent development associated with sensory feedback during critical periods of neural plasticity [17, 100, 101]. For example, results from infant sibling studies reveal differences in the visual system including the middle occipital gyrus [8] and splenium [35, 102]. Such early differences in sensory regulatory systems and the processing of environmental stimuli may have downstream, or cascading, effects that emerge as ASD symptoms [16, 17]. This also provides a springboard for the creation of a pre-symptomatic intervention that enhances (or modifies) early environmental stimuli to best match VHL-ASD infants’ vulnerabilities and lead to improved outcomes.

The processes through which a developing brain is organized require increasingly selective and efficient responses to environmental stimuli [103]. It is hypothesized that if this process (e.g., difficulty with orienting to salient information) is disrupted in particular ways, the accumulation of effects on neural circuitry could lead to the behavioral phenotype associated with ASD [104]. Within this theoretical framework, experience is implicated as one plausible causal mechanism given its essential and reciprocal role in neural development. The importance of timing and type of experience for maximizing plasticity has been well-documented in infants, such as the narrowing of face processing ability that occurs during the latter half of the first year of life [105]. Thus, modifying and enhancing an infant’s experience during the optimal time period can impact the development of neural circuitry that improves long-term cognitive and behavioral functioning [106]. An intervention would likely include ecologically valid activities tailored to the infant’s developmental challenges, and scaffolded over time.

It is likely that a theoretical framework wherein experience is posited as a mechanism for plasticity-related development in ASD could underpin many of the intervention approaches described elsewhere in this paper. For VHL-ASD infants, typical infant experiences may be insufficient to overcome genetic/biological liability. For example, the role of the gene by environment interactions has been observed to explain variance in outcomes among children adopted from Romanian orphanages, for example [107]. There is also a precedent for applying environmental enhancement-related principles to clinical practice, including interventions specific to infants [108] that target sensory-dependent plasticity via environmental modifications that include NDBIs.

Researchers have suggested [50, 109] that aspects of the complex multisensory social world are attended to and selected for differently in children who go on to develop ASD. Differences in social information encoding and processing systems could lay the foundation for altered developmental trajectories. One approach would be to modify the experiences of VHL-ASD infants to increase attention to and sampling of aspects of the social environment to optimally support developmental trajectories. Consider a scenario where a mother is feeding her infant solid food for the first time, to illustrate th e complexity of multidimensional experiences from the perspective of developing infants. The infant may enjoy the sensory experience of the food and associate certain features with positive or negative gustatory feedback. This would involve feature-based or object-based attention mechanisms of cortical learning that are available in early infancy [110], as well as item-in-context information operative as early as 6 months [111]. Differences in, for example, gustatory sensitivity might alter visual learning opportunities by virtue of reinforcement learning mechanisms. Additionally, the infant may attend to the mother’s expression, encoding any positive feedback to her eating actions. Tummeltshammer et al. [111] showed that infants as young as 8 months associate even arbitrary cues with their own mother’s reaction. Associating action with mother’s response would be an example of stimulus-action-outcome reinforcement learning [112–114]. Rewarding maternal feedback might elicit repetition of eating behaviors to receive positive rewarding feedback, eventually even without paired gustatory sensation.

The foregoing example illustrates the capacity for redundancy in learning and memory to shape human infants’ learning in multisensory events. The specific systems engaged may shape subsequent developmental trajectories, reorganize neural connections, and build learned, socially cued responses based on a domain that itself enjoyed an arbitrary initial bias (i.e., from gustatory, to object features, to social stimuli). Notably, Triesch et al. [115] demonstrated that a simple artificial agent could use reinforcement learning processes to acquire gaze following responses, which are suppressed in toddlers with ASD. Reducing the default reward value of the “caregiver’s” face, however, delayed learning of gaze-following, resembling the reduced attention-sharing of HL infants [116]. Moreover, Leekam, Lopez, & Moore (2000) [117] showed that preschoolers with ASD *can* follow gaze if extrinsically rewarded. These results, taken together, suggest that altering the experiences of VHL-ASD infants to make caregivers’ faces more motivating to watch might alter those infants’ neural networks, and yield additional experience learning associations that support a basic social skill: gaze following [118].

Targeting aspects of the infant experience that directly contribute to the development of ASD represents a plausible approach to pre-symptomatic intervention. This presupposes, however, that we currently possess sufficient knowledge of the causal pathways leading to ASD outcomes. While we have some support for the causal mechanisms [17], there is still much unknown. Addressing this knowledge gap may be expedited if multiple lines of inquiry are pursued simultaneously. For example, a systematic, experimental approach could be implemented to determine which precursor behaviors are amenable to change and whether changes in these precursors have positive downstream effects. It is likely that additional research into the target domains discussed below will be necessary, in tandem to pursuing biomarker-informed intervention targets. Similarly, we propose an equally important, but multi-faceted approach (see “Comprehensive vs. targeted approach” below) to examining effects of pre-symptomatic interventions on HL infants.

### “Setting the scene” for targeted intervention

Development of pre-symptomatic interventions should consider the benefits of targeting maximized plasticity from both a skill-oriented and a relational approach, which, historically have been separate theoretically and in application. A skill-oriented approach focuses on teaching child behaviors or skills in an adult-led and highly structured context that is often not embedded (or embedded in a highly controlled fashion) from routine daily activity contexts in order to maximize salience of instructional elements and achieve a high level of control over elements considered to be active intervention ingredients. The dyadic relational approach focuses on aspects of caregiver–child interaction, using child-responsive strategies to target social engagement and interpersonal synchrony, with the aim of supporting downstream, distal behaviors in the child. Here, we highlight the importance of integrating these conceptual approaches within pre-symptomatic interventions, where child skin enhancement is targeted within the context of relational enhancement. Any pre-symptomatic intervention during ages 6 to 12 months must consider the daily environment, expected norms, limitations, and developmental accommodations necessary for young children during this period of development as well as younger developmental ages. As a key element of young children’s daily life is the caregiver, the caregiver–child relationship must be central to any pre-symptomatic intervention.

 For human infants, the development of key caregiver–child relationships is essential for survival and the infant’s environment is greatly defined by these key relationships and associated social interactions. Relationships themselves are a product of transactional processes [119] that gradually establish a shared set of mutually understood expectations and accepted roles between participants [120]. The building blocks of these relationships rely extensively on the caregiver’s ability to actively engage the child in a manner that displays high levels of sensitive and responsive interaction patterns and to provide consistent and appropriate forms of positive affect [94]. These constructs and their associated components can be measured in a variety of contexts and provide an important framework for the design of intervention programs for VHL-ASD infants. Challenges to relationship formation, engagement, and reciprocity are particularly relevant for VHL-ASD infants. Although variable in expression, these relationship difficulties are evident in those everyday family routines and activities that require children to draw upon and integrate available developmental resources and processes to organize their behavioral patterns and pursue their goals. One major manifestation of these systems-level organizational difficulties is the reduced number of initiations that VHL-ASD infants make with their social environment. Caregivers can be supported to adapt and tune their own behaviors in order to increase the child’s number of initiations with the environment and to promote downstream relationship formation and environmental learning.

### Key intervention targets

Conceptually, pre-symptomatic intervention for ASD during the infancy period has the potential to shape infants’ developmental course such that the likelihood of later functioning and adaptation is improved in tandem with a decrease in symptoms and severity [121, 122]. Based on this line of reasoning, in a controlled pre-symptomatic ASD intervention trial, we would expect to see the trajectory of development of infants in the intervention group increasingly diverging from that of a control group across time, ideally extending beyond the end of active intervention. The intervention targets described below are separated by specific areas of development that, through modification of an infant’s environment (people, objects, etc.), might enhance skill-oriented trajectories as described, leading to improved outcomes in VHL-ASD infants. Within a relational approach described above, these targets combine direct skill learning with the foundational learning for later skill acquisition.

#### Sensory regulation

Some research indicates that HL infants who later develop ASD also show early differences in their processing of sensory information, such as displaying aversion to, over-interest in, and/or lack of expected response to various sensory inputs (e.g., visual, auditory, tactile). Three broad types of sensory reactivity patterns among children diagnosed with ASD have been widely described in the literature: hyporeactivity, hyper-reactivity, and sensory seeking [123]. There is limited research, however, to inform our understanding of the emergence of these patterns in infants who later meet criteria for ASD. Brock et al. found that regardless of the sensory reactivity patterns exhibited by preschool-aged children with ASD, the predominant pattern observed in home videos of these children as infants is hyporeactivity to sensory stimuli [124]. Wolff et al. reported that all patterns of sensory reactivity were elevated in 12-month-old HL siblings who were later diagnosed with ASD relative to those who were not [125]. Based on a prospective caregiver report, findings from that study indicated that hyper-responsivity, and sensory responsiveness involving tactile stimuli showed the greatest group difference at age 12 months [125].

Sensory regulatory patterns associated with later ASD are more stable across the second half of the first year and into the second year than is the case for social communication symptoms [126]. This observation suggests that sensory-regulatory symptoms will be less malleable to intervention than social communication symptoms, although this hypothesis has yet to be tested. Moreover, sensory regulation (and self-regulation more broadly) emerges from dyadic co-regulatory processes between infants and their caregivers [127] suggesting that interventions that target those dyadic processes may be able to alter the outcomes for infants who later develop ASD [128].

In considering sensory-regulatory targets, it is important to recognize that, although an infant may exhibit some predominant patterns of sensory arousal, reactivity to sensory stimuli can be are highly variable within infants across contexts. Therefore, these interventions will need to reflect the dynamic multi-sensory experiences of infants, which include constantly changing combinations of visual, auditory, tactile, gustatory, olfactory, and proprioceptive/vestibular sensory input. One intervention possibility is to teach caregivers to recognize the signs of sensory dysregulation (hyporeactivity, hyper-reactivity, or sensory seeking) and to assist the child in obtaining optimal regulation. In the case of hyper-arousal, caregivers could reduce multi-sensory information (e.g., minimize salient stimuli by lowering extraneous stimuli such as background noise) with the aim of reducing arousal (e.g., processing load), thereby assisting in regulation. In contrast, to treat hypo-arousal caregivers might add environmental stimuli to help the child respond appropriately (e.g., instead of calling the child’s name, touch his or her shoulder and call his or her name, thus adding tactile stimuli). These environmental modifications could be gradually decreased as the child demonstrates an increasing capacity to self-regulate.

#### Attentional biases & flexibility

Research indicates that specific experiences can tune infants’ attention to features of the environment. Numerous studies show that infants can distribute their attention in complex displays for information sampling [129]. For example, infants’ biases to attend to informative regions of own- and other-race faces are influenced by prior experiences with racially homogenous or heterogeneous faces [130], and these biases can be reversed by experimentally manipulating attentional experiences [131]. Furthermore, typically developing (TD) infants as young as 6 months will modify their attentional control during a brief intervention, through the use of gaze-contingent computer paradigms [132, 133]. Building an infant’s attentional control has potential to improve joint attention, early receptive language skills, and possibly emotion regulation [134].

Although we know that interventions can shape the information that infants’ sample from their environment, to refine sophisticated intervention, we will need naturalistic datasets comparing how VHL-ASD, HL, and LL infants experience everyday naturalistic interactions across activity contexts (e.g., play, mealtime, daily care routines, errands, etc.; see [135, 136]). Such an effort should build on recent work using mobile dense data sampling to track infant experiences in daily activities [137–139]. Such data could optimize interventions by identifying social learning input that is naturally reduced in VHL-ASD infants, and designing environmental modifications to scaffold attentional control and boost compensatory input. Goals would include, for example, teaching VHL-ASD infants, perhaps using reinforcement learning methods [113, 114], to more flexibly shift their attention between people and objects [109], and adapting their environment to enhance the reward value of social attention [115] within everyday contexts. This could facilitate VHL-ASD infants in encoding of information that supports social development. This approach will require updated behavioral measurements. Such tools might include synchronous auditory recording, mobile eye tracking, synchronized video and motion analysis, and physiological measures [137, 140]. Because these methods are under continual development, focused and sustained research efforts will be needed to obtain and analyze datasets that can yield actionable attentional scaffolding approaches for pre-symptomatic interventions.

#### Early motor skills

Infants interact with people and objects, and thus, engender self-experience, through their motor system. Evidence indicates that infants later diagnosed with ASD often exhibit early motor delays [141]. A meta-analysis focused on motor development, including data from 1233 children with ASD and 2032 with TD across the first 4 years of life, revealed that children with ASD had slower motor development than TD [141]. Early motor delays or differences are detectable in HL infants by 6 months, including atypical general movements [142, 143], postural control delays [31, 144, 145], head lag in a pull-to-sit task [31, 146], and delays in sitting [144, 147]. By 6 months of age, HL infants showed delayed grasping of items [148], reduced object exploration in free-play tasks [149, 150], and delayed bilateral hand use when retrieving an object [151]. Motor functioning at age 6 months is predictive of later communication and social functioning in HL infants [31, 141, 152]. Differences from the norm in the motor functioning of HL infants become amplified with age [141, 153, 154]. While differences in motor development have not always been disorder-specific, aberrations from typical motor development (LL) may still yield informative targets.

These results highlight that from very early on, motor delays and differences in children with ASD might impede adaptation to their environment. Even subtle disruptions or delays in early motor development likely have various cascading effects on other developmental domains and systems [155]. For example, sitting independently increases infants’ access to objects and improves positioning for exploring objects [33]. This developmental milestone thus provides rich learning opportunities that can promote developmental progress and social interaction experiences. In TD, infants’ postural attainments are associated with proximal and distal learning [156] such as early visual perception (e.g., figure–ground assignment [157] and language development [158]. Reduced object exploration may lead to attenuated exposure to object naming by others [159] and, hence, contribute to deleterious effects on language acquisition. Because early motor development is inextricably related to children’s experiences and to other developing systems, a pre-symptomatic intervention that focuses on redirecting early motor skills might improve developmental trajectories.

#### Social communication

Some social communication differences associated with later ASD emerge toward the end of the first year and early part of the second year of life [160], solidifying the need for pre-symptomatic interventions directly targeting social communication behaviors. Below, we outline several social communication behaviors that could be the focus of pre-symptomatic interventions. These are highlighted due to their early emergence and association with later ASD symptoms. The focus should be on a combination of promoting an increase in, or maintenance of, these behaviors as well as qualitatively enhancing the reciprocity of these behaviors.

**Directing communication** Pre-symptomatic interventions should seek to maintain early emerging social nonverbal communicative behaviors (e.g., gazing at faces, directed smiles) that appear to occur at the same rates, on average, in infants later diagnosed with ASD and those who are TD until around 6 months, but then decrease in frequency in infants with later ASD in subsequent months [161–163]. Other social behaviors, such as directed vocalizations, which typically emerge at around 6 months, may not increase as expected in infants with later ASD [161].

**Joint attention** The initiation of joint attention is a potentially important aspect of pre-symptomatic intervention that may lead to improved social and language outcomes. Joint attention entails the coordination of one’s own attention to an object or event with another person’s attention to the same object or event [164]. Infant responses to joint attention involve the infant following another person’s cues (e.g., shifts in gaze, pointing) to share that person’s attention to a particular object or event [165, 166]. The use of joint attention gestures rises rapidly between 9 and 18 months in TD infants as well as in infants with non-ASD neurodevelopmental disabilities, in contrast to a minimal change in infants with ASD [167]. Moreover, variability in initiating and responding to joint attention is strongly linked to social and language outcomes in children with ASD [168–170]. Importantly, joint attention behaviors can be trained in young children with ASD [171, 172].

**Joint engagement** As a dyadic target for this population, interventions should aim to promote joint engagement between infants and their caregivers. Coordinated joint engagement, a state during which a child actively attends to a caregiver as well as an object, occurs less frequently and for limited periods of time in children with ASD [173, 174]. In contrast, supported joint engagement, a state in which children focus on the same objects and events of interest as a caregiver but do not overtly attend to the caregiver [175], can be scaffolded by caregivers following their child’s focus of attention and joining in the child’s activity. This increases the frequency and extends the time that children spend in these states. The amount of time young children with ASD spend in supported joint engagement is a significant predictor of their word learning [173, 174] and verb learning specifically [176]. Thus, increasing the frequency and length of supported joint engagement states as a dyadic target is a strong candidate for pre-symptomatic interventions, particularly those that fully or partially rely on caregiver participation.

### Designing pre-symptomatic interventions

Research into early intervention programs for children with ASD has provided some guidance about the most effective techniques for the development of a pre-symptomatic intervention. Specific decisions about who implements the intervention (e.g., caregivers vs. clinicians), where the intervention should take place, and what materials should be used all determine the ecological validity of an intervention and can aid in promoting generalization of skills. Although detailed exploration of all these nuances will be necessary, such details are beyond the scope of the current article. Ongoing research will provide more guidance about the critical components needed in a pre-symptomatic intervention, variable or optional aspects of an intervention, and factors that should be tailored to individual differences and familial resources.

### Ongoing assessment and monitoring

Ongoing assessment and monitoring are necessary to establish the efficacy of any intervention. Given that some symptoms emerge in their earliest forms during the 6-to-12-month period, a combination of measures that both monitor symptoms and other changes over time may be useful. Utilizing measures that leverage video-based data will provide the opportunity to make more nuanced, contextually informed decisions about the targeted behavior than are possible with typical broader measures of development (e.g., Mullen Scales of Early Learning (MSEL) [177]). Video-based data allows for quantitative and qualitative assessments of both children and their parents, dyadic behaviors, and engagement [178, 179]. Video data files can be utilized multiple times to derive—or to innovate—various behavioral measures. For example, these data can be used to identify sensory reactivity to auditory, visual, or audiovisual experiences. The Manchester Assessment of Caregiver-Infant Interactions (MACI) was designed for use during the ASD prodromal period with infants at heightened likelihood of developing ASD. It has been used as an outcome measure in two pre-symptomatic RCTs [70, 78, 180]. The Brief Observation of Social Communication Change (BOSCC; [181]) offers an option for tracking intervention-associated changes in ASD symptomology in children, though this has not been evaluated in children under 12 months, or in those without early emerging symptoms. It is clear that new behavioral measures will need to be developed and standardized in order to evaluate the efficacy of pre-symptomatic interventions.

Other standardized measures that utilize either caregiver report or direct observation may also be useful. The MSEL and the Bayley Scales of Infant–Toddler Development (BSID [182]) both provide standardized norms across multiple developmental domains. The Autism Observation Scale for Infants (AOSI [183]) and the Communication and Symbolic Behavior Scales Developmental Profile Behavior Sample (CSBS DP; [184]) are examiner-administered measures involving presentation of semi-structured prompts to elicit targeted behaviors. The MacArthur-Bates Communicative Inventory (MCDI), the Autism Parent Screen for Infants (APSI; [185]), and the Parent Observation of Early Markers Scale (POEMS; [186]) all require or offer the opportunity for caregiver reports. Although these measures often have been used to quantify outcomes, such global metrics might not be effective at capturing the nuanced proximal changes over time in pre-symptomatic interventions. Thus, a combination of rich, longitudinal behavioral records, and periodic “benchmark” standardized test scores might prove most effective in tracking the emergence of symptomatic behavioral traits.

### Caregiver role

#### Family adaptive functioning

Innovative and paradigm-shifting efforts to develop pre-symptomatic interventions for VHL-ASD infants should not lead us to overlook the most straightforward opportunities that we have available to support development in this population. The pre-symptomatic window during infancy is a time in which caregiver coaching and education can be implemented to support and, when necessary, to improve family adaptive functioning for the benefit of the VHL-ASD infant and all family members.

The role caregivers play in families of children with developmental disabilities can be understood in the larger context of family adaptive functioning; the range of activities that families, usually caregivers, conduct on behalf of their children with disabilities (e.g., family-orchestrated child experiences, caregiver–child interaction, child health and safety functions [94, 187]. This model addresses the fact that caregivers vary in terms of personal characteristics (mental health, coping ability) and resources (child development knowledge, social support, financial). It is important to note that parents themselves may have ASD, the broader ASD phenotype or another child with ASD. These family environments may have implications for intervention efficacy. As such, a reflective coaching approach may be beneficial, as it ensures that families’ views and priorities are considered. Any caregiver coaching should be tailored to the caregiver’s learning style and pace [122, 188]. These factors combine to influence caregiver readiness to engage in pre-symptomatic identification and intervention. The pre-symptomatic window is an opportunity to work with caregivers, assess their readiness, and intervene to ensure caregivers have the resources to meet the challenges that emerging symptoms will pose. Pre-symptomatic interventions and pre-symptomatic assessment and monitoring protocols should be developed to promote family adaptive functioning, caregiver advocacy skills, knowledge of infant and toddler development, and caregiver sense of efficacy [189].

#### Caregivers as interventionists

To date, there have been only a handful of interventions using a caregiver-mediated approach (as described above in “Preliminary studies of pre-symptomatic intervention for ASD”) including both small pilot studies [122, 190–192] and several larger RCTs (i.e., 54–103 families; [76, 77, 79, 80, 193, 194]). As discussed above, these studies have shown few main effects on child outcomes, and effects identified have not been seen on primary outcome measures. Yet changes in caregiver responsiveness have been found to mediate improvements in child behavior in some RCTs [69, 77, 195]. This highlights that using a caregiver-mediated approach provides an opportunity to modify caregiver behavior that can have subsequent impacts on child behavior.

It is important to note that focusing on the behavior of caregivers as a mechanism of intervention response is not because there is any atypical or problematic caregiver behavior in VHL-ASD families; indeed, caregivers of VHL-ASD children likely are just as sensitive and responsive as caregivers of TD children, and provide similar environments for their children [196, 197]. However, it is possible that the VHL-ASD child’s vulnerabilities require enhanced, more explicitly targeted caregiver responses, or modulations of stimuli during caregiver–child interactions, above and beyond what is necessary in non-VHL-ASD development.

The effects of caregiver-mediated interventions are variable across families. Factors that could affect caregivers’ uptake of an intervention include how well the intervention techniques are taught; intensity of other time demands (e.g., job-related); number and demands of other children or family members with health problems or disabilities; presence and availability of a parenting partner; health status of the caregivers themselves, including mental health; alignment between intervention and caregivers’ cultural perspectives; supports available to caregivers besides the intervention itself; caregivers’ levels of concern about the child; and many more [198].

One key challenge in studying caregiver-mediated interventions is measuring the quality or dosage of intervention provided by caregivers, given that the general goal of these interventions is to impact caregivers’ daily interactions with their children. Caregiver implementation of the intervention can vary—both with respect to the fidelity of implementation (e.g., responsiveness to the child’s interests or other cues) and with the dosage of delivery (e.g., frequency, duration, and intensity of use within daily interactions). Hence, researchers are restricted in evaluating what would be an optimal dosage of intervention. For example, investigations are needed to define the quantity of synchronous responding that would be sufficient to impact child outcomes [199].

Caregivers of children with ASD often experience more stress than caregivers of TD and developmentally delayed children [200, 201]. Adding the burden of acting as an interventionist has the potential to further increase stress, which should be considered and monitored as interventions are developed and implemented. Research has shown that there is a relationship between caregiver stress and caregivers’ feelings of efficacy during an intervention [202].

Consistent with foundational work on clinical intervention for young children with ASD or at HL for ASD, the development of caregiver-mediated models of pre-symptomatic interventions for VHL-ASD infants should:

1. Target the behaviors described above (motor skills, sensory regulation, attentional biases and flexibility, social communication) and be embedded within the context of establishing and enhancing foundational relationships
2. Take place in the child’s natural environment, unless the family is in a situation that precludes it, or in some instances where community-based implementation may be more feasible (e.g., in a childcare setting). It bears emphasizing that opportunities to embed learning into everyday routines can promote family uptake and thus increase opportunities for practice. Clinicians and family caregivers should collaborate in determining each family’s unique needs, and implementing interventions that best meet those needs
3. Model a collaborative and supportive approach with families as this may hold particular promise for sustaining parental buy-in and engagement, and thus mediating long-term positive change
4. Build upon families’ strengths and interests, resulting in greater adoption of the strategies
5. Promote emotionally positive caregiver–child interactions, as found in some infant mental health approaches (e.g., PFR; [98])
6. Promote equity of access, feasibility, and cultural sensitivity in the program design. Feasible programs ensure that strategies can be learned by parents or other key caregivers in a short period of time, and implemented with fidelity by families with different levels of proficiency in the language of instruction, low literacy, and a wide range of educational and socioeconomic backgrounds. Culturally sensitive practices must be embedded into program design to ensure that a particular approach is both feasible and culturally meaningful, or can be adapted as necessary, to accommodate a wide range of culturally and linguistically diverse families.
7. Scaffold caregivers’ skills and allow them to experience success. This strategy is likely to be most effective in fostering self-efficacy and empowerment, which may support ongoing engagement and mitigate stress

### Clinician role

#### Clinician as coach and/or interventionist

A key line of future investigation is the careful design and evaluation of the caregiver coaching approach [203]. Focused attention and training in adult learning methods, and evaluation of specific coaching strategies that are best equipped to support the diverse range of caregivers of VHL-ASD infants, are essential. As described above, early intervention studies that have predominantly utilized caregiver-mediated models have shown small to modest effects, though this provides the opportunity for further development and implementation of intervention during a more sensitive window. It is possible that intervention delivery solely by a caregiver is insufficient to drive substantive and long-lasting changes in infant development, at least for some children. Given the limitations of caregiver-mediated intervention discussed above, adjunct delivery by clinicians could offer some potential advantages to the child, and may also improve the interpretability of results. For example, some variables may be easier to control or standardize in clinician-delivered programs, such as intervention dosage and fidelity of implementation. For example, implementation fidelity can be ensured by requiring that clinicians achieve implementation fidelity before (and throughout) providing the intervention, which may not always be possible with parent-mediated interventions. As we design and test pre-symptomatic interventions for VHL-ASD infants, we may find that some targeted outcomes are only possible if addressed using technically precise and complex intervention strategies that are difficult to efficiently promote in caregivers who may not have the specialized expertise.

In research piloting an innovative pre-emptive intervention, investigators should engage stakeholders (especially caregivers) in designing and testing flexible models of direct services incorporating collaborative decision-making with caregivers. The unique role of the clinician may differ from family to family, but might include a combination of caregiver support, coaching, and direct delivery of intervention strategies.

### Comprehensive vs. targeted approach

Considering the variety of potential targets and implementation strategies for a pre-symptomatic intervention, this manuscript provides only an initial guide and not a detailed plan to direct next research steps. As the field grows and new research emerges to guide the development of a pre-symptomatic intervention, one strategy would be to develop an intervention by the comprehensive approach, combining aspects of all, or many, of the components described above. For example, an approach that combines both caregiver and clinician components, and developmental outcome targets that span a range of developmental domains (e.g., sensory, motor, attentional, and social) might be necessary to promote child adaptations that lead to the best outcomes. However, such a broad range would only be valuable if followed up with well-articulated examination of “active ingredients” or key components that were most impactful to the child and family. Another concern of such a broad approach is that it might render each interventional component too diffuse (given, e.g., dosage–time limitations, which are particularly salient in the early years when young children’s availability for learning may occur in brief spurts) to yield sufficient impact on developmental trajectories. In addition, a broad approach may be too labor intensive, costly, and burdensome to families and service systems to be feasible, particularly in the light of “probable” likelihood of ASD rather than a confirmed diagnosis. Research might reveal that pre-symptomatic interventions should prioritize certain implementation strategies or developmental targets in initial attempts and modify these choices based on results of ongoing intervention studies.

In contrast to a comprehensive approach, it may be more feasible to focus on one aspect or one target in an intervention. In a targeted intervention approach, it may be easier to measure improvements in highly constrained domains. Yet, the nature of outcomes that researchers are seeking are broad and span a range of developmental categories. Ideally, intervention outcomes will include improvements across a broad range of major developmental milestones, as well as reduction in the severity or functional impact of later ASD symptoms. Realistically, however, the cost of sufficiently powered studies of VHL-ASD infants will limit the number of focused intervention studies that can be undertaken to make entirely research-informed decisions, so some studies using comprehensive approaches are likely needed for initial tests of efficacy. Put differently, early pre-symptomatic intervention attempts will need to strike the ideal balance between being highly focused on a specific target or population but lacking in context, or underpowered and too diffuse to draw clear causal inferences. However, the cumulative research efforts can be coordinated to eventually elucidate which targets and methods are most advantageous, ultimately providing sufficient data to support a large-scale, evidence-based, focused RCTs.

Current early intervention strategies provide guidance on methods for intervening in social communication and motor skills, but less guidance about strategies for intervening in targets such as visual attention and sensory regulation. While providing detailed methodology to “treat” all proposed targets would be helpful, the current literature does not provide sufficient evidence to suggest more than this broad description of conceptual targets. That said, we hope that this framework will catalyze more research to determine effective approaches to address the latter targets. At this point, however, researchers will need to develop novel methods for fostering sensory regulation and attentional control/flexibility in infants. This is especially true given the limited effectiveness thus far of interventions for social communication targets. Efforts will require extensive characterization of sensory regulation and visual attention differences, at different ages, between VHL-ASD and LL infants in order to define intervention sequences. Then, methods for altering disrupted trajectories can be tested. However, because this research strategy would necessarily require a great deal of time, ideally these various efforts would occur in parallel so that pre-symptomatic interventions can be progressively matched or optimized to evidence about traits and diagnostic outcomes of VHL-ASD infants.

### Considerations in the development of pre-symptomatic interventions

#### Ethical considerations

In the development of any pre-symptomatic intervention, a bioethical framework should be used from the beginning of this effort to evaluate the ethical, legal, and social implications (ELSI) of pre-symptomatic identification and intervention [204]. To ensure that pre-symptomatic interventions are feasible, accessible, and desirable, several ethical benchmarks should be considered [205]. These benchmarks include *collaborative partnerships*, *maximizing social value*, and a *favorable risk benefit ratio*. Establishing *collaborative partnerships* with ASD individuals and their families will be essential to ensure that pre-symptomatic interventions will ultimately be integrated into community care in meaningful, culturally and individually relevant, and sustainable way [204, 206]. We note that several underserved populations generally face a lack of representation in research, and this gap in the research should be remediated. For example, in a large study of black children with ASD, access to diagnostic assessments and subsequent care was significantly delayed compared with non-Hispanic white children [207]. Collaborative partnerships should be cultivated prior to pre-symptomatic intervention development, during pre-symptomatic intervention implementation, and following completion of any interventions. In developing this novel approach, there is also the opportunity to incorporate the perspective of autistic adults who have the lived experience of ASD and participation in ASD intervention. The neurodiversity perspective provides an opportunity to conceptualize pre-symptomatic intervention as a way to support an infant whose brain processes information differently and whose development of ASD symptoms is a result of adaptation to his or her environment in light of these processing differences. Community involvement is key for researchers using this proposed agenda as a foundation for the development of a pre-symptomatic intervention.

The potential *social value* of a pre-symptomatic intervention has been highlighted throughout this article. We assume that pre-symptomatic interventions targeting the developmental window before 12 months of age, when the brain is most malleable, can theoretically yield the best outcomes. This suggests that, with pre-symptomatic intervention, more ASD individuals will be able to participate in the community, communicate effectively, and have higher quality of life; conversely, fewer will require costly, time-consuming, and burdensome long-term supports. This also has the potential to provide caregivers with a better understanding and acceptance of, as well as adaptation to, the differences these infants may demonstrate. The social value of this is immeasurable.

Although the potential value of a pre-symptomatic intervention is high, the actual benefits are yet unknown. To truly characterize any benefits of a pre-symptomatic intervention, RCTs will be necessary. However, this will require that part of the sample, with informed consent, is randomly assigned to an intervention-as-usual condition. Researchers should be prepared to closely monitor the benefits of a pre-symptomatic intervention, and, if obvious benefits are noted, re-evaluate the plan to withhold a superior intervention from the control group or groups. One possibility is to begin with waitlist control designs, wherein the control group receives the intervention after a waiting period. While this approach may lead to challenges in interpretation of long-term outcomes, it does mitigate any short-term ethical challenges. Flexibility in study design is not standard in traditional RCT designs, so the field may benefit from attention to research designs that foster flexibility such as SMART designs.

Changes in development and function might not be the only benefit of pre-symptomatic intervention outcomes to consider for individuals with ASD and their families, both in terms of potential benefits and risks. There is evidence that caregivers value learning about their child’s health, even when direct intervention options are limited [208, 209]. Therefore, providing information to a family about their child’s likelihood status for ASD may be beneficial, regardless of the efficacy of pre-symptomatic interventions. This may give families opportunities to prepare for the future in other ways, such as by moving closer to supportive resources [210]. Conversely, some caregivers may find it difficult to learn of their child’s elevated likelihood of ASD, and potentially seek out unfounded, even potentially dangerous, interventions [211]. This reaction might be more common among caregivers assigned to a control condition. It will be essential for researchers and providers of pre-symptomatic interventions to be well-versed in informed consent processes and medical counseling techniques and prepared to provide resources to benefit caregivers’ family mental health needs. Studies are needed to determine how to provide information in a manner that caregivers find engaging, empowering, and motivating.

Some caregivers of VHL-ASD children, even those enrolled in infant sibling studies (over 40% in one study), may choose not to engage in pre-symptomatic intervention [212]. It is critical to understand the aspects of pre-symptomatic identification and intervention that are valued by caregivers and, equally importantly, the aspects that raise caregivers’ concerns. The impact of intervention demands related to dosage (hours per week), format (group, individual, or caregiver-delivered), and setting (remote/telehealth, home-based, or center-based) on caregivers’ decision-making must be systematically evaluated (e.g., to understand the effect these features have on caregiver decision-making). Caregivers from different communities, with different socioeconomic and cultural backgrounds, may vary in their responses to pre-symptomatic identification and interventions for a variety of complex reasons that are, as yet, not well understood [213].

#### Individualization vs systematic implementation

One challenge posed by pre-symptomatic intervention is how to adapt to differing baseline abilities and behaviors of individual infants and caregivers. VHL-ASD infants are likely to manifest an array of differences from TD infants (who themselves can be quite variable) across numerous early markers and characteristics. For example, one infant might have typical visual attention but gross motor delays; another infant might have significant visual attention alterations but be only mildly delayed in motor benchmarks. Similarly, the pivotal developmental targets of an intervention will need to be closely linked with a child’s ability level. For example, imitation skills may be more appropriate to teach a developmentally aged 6-month-old than a developmentally aged 18-month-old. Thus, intervention must be tailored to the developmental level and skill profile of each infant.

Perhaps the most feasible tactic is to develop interventions that carefully delineate a set of globally relevant procedures that are taught to caregivers/clinicians. Specific targets may need to be taught in unique ways but with principles that are consistent and relevant across domains such as reading the infant’s cues, noticing their focus of attention, attunement to their state of arousal, awareness of their moment-by-moment motivation, and provision of structured opportunities (e.g., to request, to respond, to enact, to attend). Together with the guiding principles described above, these considerations may provide a useful framework for developing interventions that are both individualized and sufficiently consistent to meet the needs of VHL-ASD infants. Nevertheless, the challenge of individualization will need to be considered as research into pre-symptomatic interventions proceed.

## Summary and conclusions

The overarching goal of this paper is to provide a summary of the research needs and theoretical bases underlying the development of pre-symptomatic interventions for VHL-ASD infants. As the capacity to use behavioral and biological markers to identify ASD likelihood improves, research must equally progress in identifying intervention strategies for study in VHL-ASD infants and, if proven efficacious, to maximize the likelihood that such services are offered to families. Several areas of focus for pre-symptomatic interventions are proposed, including scaffolding of social communicative behaviors and focusing on disruptions to more basic skills (e.g., motor skill, attention, and sensory regulation) that may lead to the cascading development of ASD symptoms. These suggestions are based on growing evidence of behavioral precursors to ASD symptoms in (primarily) familial-likelihood samples. We frame these suggested targets within the “adaptation as development” theory [50]. This theory emphasizes that each child has the inherent capacity for his or her best developmental trajectory. This developmental trajectory, however, is constrained by congenital phenotypes and tendencies, and by the individual’s progressive history of interactions with a dynamic environment. Environmental modifications (e.g., caregiver behaviors) can be tailored to promote perceptual and motor trajectories in a given child that will lead that child into more adaptive social, communicative, and cognitive trajectories. Although not discussed here, evidence-based pharmacological or other medical interventions might further enhance intervention effectiveness if implemented early in development [87, 89].

While the development of any intervention involves the consideration of many complex factors, it is beyond the scope of this work to highlight all of the potential barriers to intervention. Nevertheless, we acknowledge that these barriers are significant, including the immense variability present in ASD symptomology and trajectories due to etiologic heterogeneity and variation in experience, feasibility and accessibility of intervention, measurement of outcomes, and the lack of substantive research on the topic of pre-symptomatic intervention for ASD. While we do not address these points, they are critical concerns that will need to be accounted for in study designs.

The first few years of a child’s life are often stressful and demanding for caregivers, and the proposed interventions discussed in this paper may not be feasible for all families (e.g., those facing systemic barriers to access). It is important to address the question of cost, as well as the stress that early identification may cause, especially in the absence of a clinically testable intervention. Additionally, children may receive a false-positive VHL-ASD designation, further increasing family stress. Furthermore, it is important to note the differences between research goals and clinical practice; the current average age of an ASD diagnosis is 4 years [20], which naturally limits the feasibility of early intervention, especially during the time period we are advocating for. While the infrastructural deficits limiting the feasibility of pre-symptomatic intervention are beyond the scope of this work, they must also be researched and formalized.

Despite the significant barriers to pre-symptomatic intervention that remain to be addressed, this work is a critical first step toward developing a conceptual framework to intervene during the pre-symptomatic period, before the earliest age of diagnoses of ASD, to alter trajectories toward enhanced quality of life, improved communication, and more supportive family and community involvement. As this work highlights, there is much to be done across many areas to advance the development of a pre-symptomatic intervention, but the potential rewards of intervening during a more sensitive window are high. This is an exciting time when our research has the potential to address a public health challenge and ultimately, to improve the quality of life of children with ASD and their families. 

## Data Availability

Not applicable
